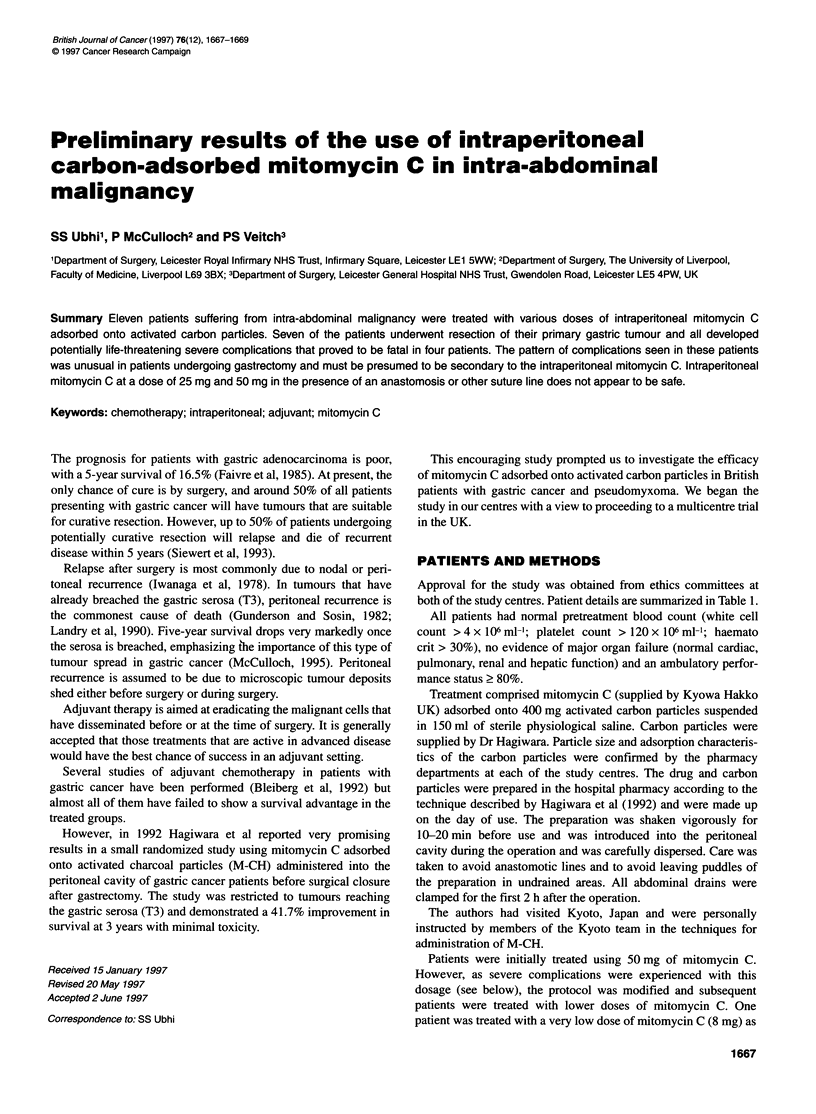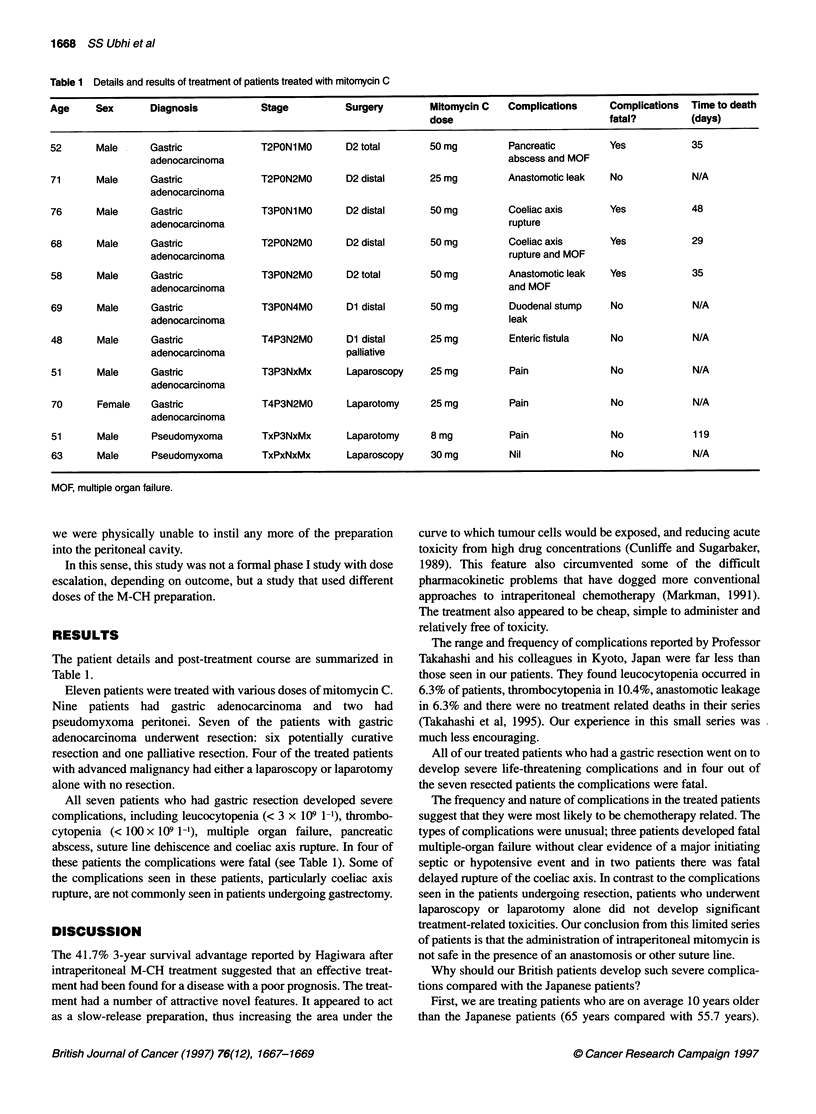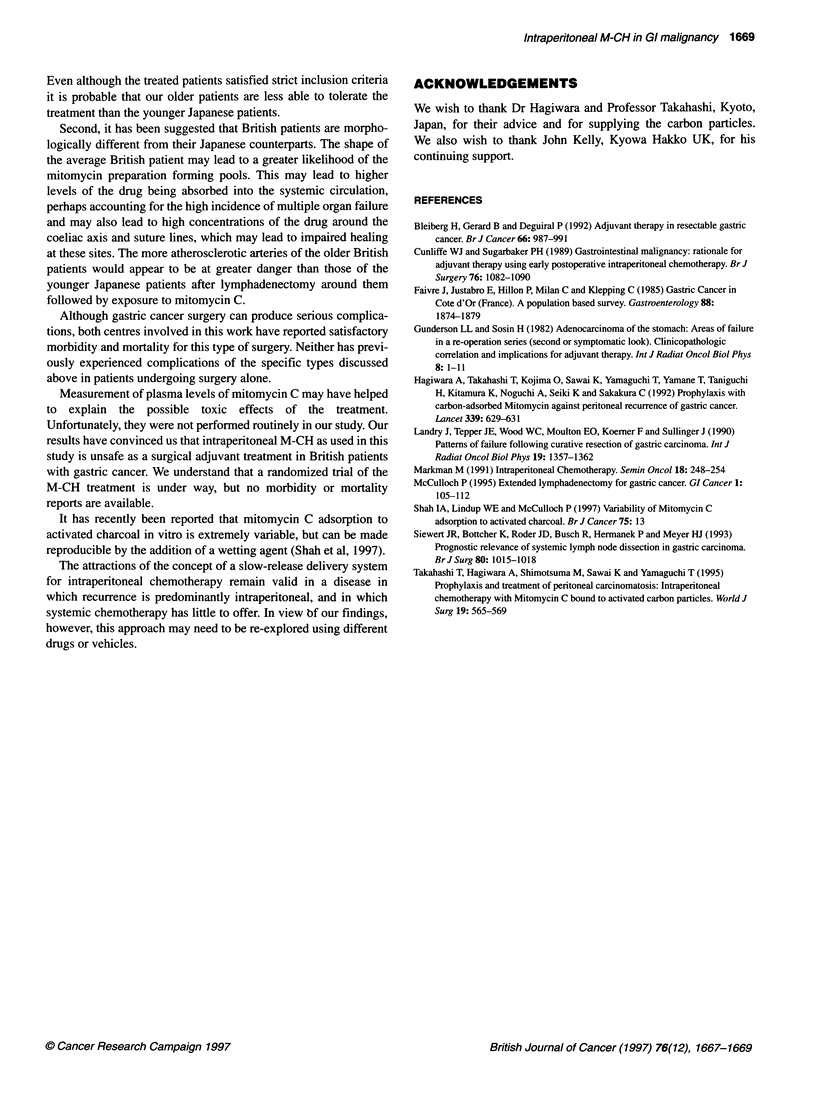# Preliminary results of the use of intraperitoneal carbon-adsorbed mitomycin C in intra-abdominal malignancy.

**DOI:** 10.1038/bjc.1997.615

**Published:** 1997

**Authors:** S. S. Ubhi, P. McCulloch, P. S. Veitch

**Affiliations:** Department of Surgery, Leicester Royal Infirmary NHS Trust, UK.

## Abstract

Eleven patients suffering from intra-abdominal malignancy were treated with various doses of intraperitoneal mitomycin C adsorbed onto activated carbon particles. Seven of the patients underwent resection of their primary gastric tumour and all developed potentially life-threatening severe complications that proved to be fatal in four patients. The pattern of complications seen in these patients was unusual in patients undergoing gastrectomy and must be presumed to be secondary to the intraperitoneal mitomycin C. Intraperitoneal mitomycin C at a dose of 25 mg and 50 mg in the presence of an anastomosis or other suture line does not appear to be safe.


British Journal of Cancer (1997) 76(12), 1667–1669
© 1997 Cancer Research Campaign

### Preliminary results of the use of intraperitoneal carbon-adsorbed mitomycin C in intra-abdominal malignancy

**SS Ubhi[1], P McCulloch[2] and PS Veitch[3]**

[1]Department of Surgery, Leicester Royal Infirmary NHS Trust, Infirmary Square, Leicester LE1 5WW; [2]Department of Surgery, The University of Liverpool, Faculty of Medicine, Liverpool L69 3BX; [3]Department of Surgery, Leicester General Hospital NHS Trust, Gwendolen Road, Leicester LE5 4PW, UK

**Summary** Eleven patients suffering from intra-abdominal malignancy were treated with various doses of intraperitoneal mitomycin C adsorbed onto activated carbon particles. Seven of the patients underwent resection of their primary gastric tumour and all developed potentially life-threatening severe complications that proved to be fatal in four patients. The pattern of complications seen in these patients was unusual in patients undergoing gastrectomy and must be presumed to be secondary to the intraperitoneal mitomycin C. Intraperitoneal mitomycin C at a dose of 25 mg and 50 mg in the presence of an anastomosis or other suture line does not appear to be safe.

**Keywords:** chemotherapy; intraperitoneal; adjuvant; mitomycin C

The prognosis for patients with gastric adenocarcinoma is poor, with a 5-year survival of 16.5% (Faivre et al, 1985). At present, the only chance of cure is by surgery, and around 50% of all patients presenting with gastric cancer will have tumours that are suitable for curative resection. However, up to 50% of patients undergoing potentially curative resection will relapse and die of recurrent disease within 5 years (Siewert et al, 1993).

Relapse after surgery is most commonly due to nodal or peritoneal recurrence (Iwanaga et al, 1978). In tumours that have already breached the gastric serosa (T3), peritoneal recurrence is the commonest cause of death (Gunderson and Sosin, 1982; Landry et al, 1990). Five-year survival drops very markedly once the serosa is breached, emphasizing the importance of this type of tumour spread in gastric cancer (McCulloch, 1995). Peritoneal recurrence is assumed to be due to microscopic tumour deposits shed either before surgery or during surgery.

Adjuvant therapy is aimed at eradicating the malignant cells that have disseminated before or at the time of surgery. It is generally accepted that those treatments that are active in advanced disease would have the best chance of success in an adjuvant setting.

Several studies of adjuvant chemotherapy in patients with gastric cancer have been performed (Bleiberg et al, 1992) but almost all of them have failed to show a survival advantage in the treated groups.

However, in 1992 Hagiwara et al reported very promising results in a small randomized study using mitomycin C adsorbed onto activated charcoal particles (M-CH) administered into the peritoneal cavity of gastric cancer patients before surgical closure after gastrectomy. The study was restricted to tumours reaching the gastric serosa (T3) and demonstrated a 41.7% improvement in survival at 3 years with minimal toxicity.

*Received 15 January 1997*
*Revised 20 May 1997*
*Accepted 2 June 1997*

*Correspondence to:* SS Ubhi

This encouraging study prompted us to investigate the efficacy of mitomycin C adsorbed onto activated carbon particles in British patients with gastric cancer and pseudomyxoma. We began the study in our centres with a view to proceeding to a multicentre trial in the UK.

### PATIENTS AND METHODS

Approval for the study was obtained from ethics committees at both of the study centres. Patient details are summarized in Table 1.

All patients had normal pretreatment blood count (white cell count $> 4 \times 10^6$ ml$^{-1}$; platelet count $> 120 \times 10^6$ ml$^{-1}$; haemato crit > 30%), no evidence of major organ failure (normal cardiac, pulmonary, renal and hepatic function) and an ambulatory performance status ≥ 80%.

Treatment comprised mitomycin C (supplied by Kyowa Hakko UK) adsorbed onto 400 mg activated carbon particles suspended in 150 ml of sterile physiological saline. Carbon particles were supplied by Dr Hagiwara. Particle size and adsorption characteristics of the carbon particles were confirmed by the pharmacy departments at each of the study centres. The drug and carbon particles were prepared in the hospital pharmacy according to the technique described by Hagiwara et al (1992) and were made up on the day of use. The preparation was shaken vigorously for 10–20 min before use and was introduced into the peritoneal cavity during the operation and was carefully dispersed. Care was taken to avoid anastomotic lines and to avoid leaving puddles of the preparation in undrained areas. All abdominal drains were clamped for the first 2 h after the operation.

The authors had visited Kyoto, Japan and were personally instructed by members of the Kyoto team in the techniques for administration of M-CH.

Patients were initially treated using 50 mg of mitomycin C. However, as severe complications were experienced with this dosage (see below), the protocol was modified and subsequent patients were treated with lower doses of mitomycin C. One patient was treated with a very low dose of mitomycin C (8 mg) as

**Table 1** Details and results of treatment of patients treated with mitomycin C

| Age | Sex | Diagnosis | Stage | Surgery | Mitomycin C dose | Complications | Complications fatal? | Time to death (days) |
|---|---|---|---|---|---|---|---|---|
| 52 | Male | Gastric adenocarcinoma | T2P0N1M0 | D2 total | 50 mg | Pancreatic abscess and MOF | Yes | 35 |
| 71 | Male | Gastric adenocarcinoma | T2P0N2M0 | D2 distal | 25 mg | Anastomotic leak | No | N/A |
| 76 | Male | Gastric adenocarcinoma | T3P0N1M0 | D2 distal | 50 mg | Coeliac axis rupture | Yes | 48 |
| 68 | Male | Gastric adenocarcinoma | T2P0N2M0 | D2 distal | 50 mg | Coeliac axis rupture and MOF | Yes | 29 |
| 58 | Male | Gastric adenocarcinoma | T3P0N2M0 | D2 total | 50 mg | Anastomotic leak and MOF | Yes | 35 |
| 69 | Male | Gastric adenocarcinoma | T3P0N4M0 | D1 distal | 50 mg | Duodenal stump leak | No | N/A |
| 48 | Male | Gastric adenocarcinoma | T4P3N2M0 | D1 distal palliative | 25 mg | Enteric fistula | No | N/A |
| 51 | Male | Gastric adenocarcinoma | T3P3NxMx | Laparoscopy | 25 mg | Pain | No | N/A |
| 70 | Female | Gastric adenocarcinoma | T4P3N2M0 | Laparotomy | 25 mg | Pain | No | N/A |
| 51 | Male | Pseudomyxoma | TxP3NxMx | Laparotomy | 8 mg | Pain | No | 119 |
| 63 | Male | Pseudomyxoma | TxPxNxMx | Laparoscopy | 30 mg | Nil | No | N/A |

MOF, multiple organ failure.

we were physically unable to instil any more of the preparation into the peritoneal cavity.

In this sense, this study was not a formal phase I study with dose escalation, depending on outcome, but a study that used different doses of the M-CH preparation.

### RESULTS

The patient details and post-treatment course are summarized in Table 1.

Eleven patients were treated with various doses of mitomycin C. Nine patients had gastric adenocarcinoma and two had pseudomyxoma peritonei. Seven of the patients with gastric adenocarcinoma underwent resection: six potentially curative resection and one palliative resection. Four of the treated patients with advanced malignancy had either a laparoscopy or laparotomy alone with no resection.

All seven patients who had gastric resection developed severe complications, including leucocytopenia ($< 3 \times 10^9\ l^{-1}$), thrombocytopenia ($< 100 \times 10^9\ l^{-1}$), multiple organ failure, pancreatic abscess, suture line dehiscence and coeliac axis rupture. In four of these patients the complications were fatal (see Table 1). Some of the complications seen in these patients, particularly coeliac axis rupture, are not commonly seen in patients undergoing gastrectomy.

### DISCUSSION

The 41.7% 3-year survival advantage reported by Hagiwara after intraperitoneal M-CH treatment suggested that an effective treatment had been found for a disease with a poor prognosis. The treatment had a number of attractive novel features. It appeared to act as a slow-release preparation, thus increasing the area under the curve to which tumour cells would be exposed, and reducing acute toxicity from high drug concentrations (Cunliffe and Sugarbaker, 1989). This feature also circumvented some of the difficult pharmacokinetic problems that have dogged more conventional approaches to intraperitoneal chemotherapy (Markman, 1991). The treatment also appeared to be cheap, simple to administer and relatively free of toxicity.

The range and frequency of complications reported by Professor Takahashi and his colleagues in Kyoto, Japan were far less than those seen in our patients. They found leucocytopenia occurred in 6.3% of patients, thrombocytopenia in 10.4%, anastomotic leakage in 6.3% and there were no treatment related deaths in their series (Takahashi et al, 1995). Our experience in this small series was much less encouraging.

All of our treated patients who had a gastric resection went on to develop severe life-threatening complications and in four out of the seven resected patients the complications were fatal.

The frequency and nature of complications in the treated patients suggest that they were most likely to be chemotherapy related. The types of complications were unusual; three patients developed fatal multiple-organ failure without clear evidence of a major initiating septic or hypotensive event and in two patients there was fatal delayed rupture of the coeliac axis. In contrast to the complications seen in the patients undergoing resection, patients who underwent laparoscopy or laparotomy alone did not develop significant treatment-related toxicities. Our conclusion from this limited series of patients is that the administration of intraperitoneal mitomycin is not safe in the presence of an anastomosis or other suture line.

Why should our British patients develop such severe complications compared with the Japanese patients?

First, we are treating patients who are on average 10 years older than the Japanese patients (65 years compared with 55.7 years).

© Cancer Research Campaign 1997

Even although the treated patients satisfied strict inclusion criteria it is probable that our older patients are less able to tolerate the treatment than the younger Japanese patients.

Second, it has been suggested that British patients are morphologically different from their Japanese counterparts. The shape of the average British patient may lead to a greater likelihood of the mitomycin preparation forming pools. This may lead to higher levels of the drug being absorbed into the systemic circulation, perhaps accounting for the high incidence of multiple organ failure and may also lead to high concentrations of the drug around the coeliac axis and suture lines, which may lead to impaired healing at these sites. The more atherosclerotic arteries of the older British patients would appear to be at greater danger than those of the younger Japanese patients after lymphadenectomy around them followed by exposure to mitomycin C.

Although gastric cancer surgery can produce serious complications, both centres involved in this work have reported satisfactory morbidity and mortality for this type of surgery. Neither has previously experienced complications of the specific types discussed above in patients undergoing surgery alone.

Measurement of plasma levels of mitomycin C may have helped to explain the possible toxic effects of the treatment. Unfortunately, they were not performed routinely in our study. Our results have convinced us that intraperitoneal M-CH as used in this study is unsafe as a surgical adjuvant treatment in British patients with gastric cancer. We understand that a randomized trial of the M-CH treatment is under way, but no morbidity or mortality reports are available.

It has recently been reported that mitomycin C adsorption to activated charcoal in vitro is extremely variable, but can be made reproducible by the addition of a wetting agent (Shah et al, 1997).

The attractions of the concept of a slow-release delivery system for intraperitoneal chemotherapy remain valid in a disease in which recurrence is predominantly intraperitoneal, and in which systemic chemotherapy has little to offer. In view of our findings, however, this approach may need to be re-explored using different drugs or vehicles.

### ACKNOWLEDGEMENTS

We wish to thank Dr Hagiwara and Professor Takahashi, Kyoto, Japan, for their advice and for supplying the carbon particles. We also wish to thank John Kelly, Kyowa Hakko UK, for his continuing support.

### REFERENCES

Bleiberg H, Gerard B and Deguiral P (1992) Adjuvant therapy in resectable gastric cancer. *Br J Cancer* **66:** 987–991

Cunliffe WJ and Sugarbaker PH (1989) Gastrointestinal malignancy: rationale for adjuvant therapy using early postoperative intraperitoneal chemotherapy. *Br J Surgery* **76:** 1082–1090

Faivre J, Justabro E, Hillon P, Milan C and Klepping C (1985) Gastric Cancer in Cote d'Or (France). A population based survey. *Gastroenterology* **88:** 1874–1879

Gunderson LL and Sosin H (1982) Adenocarcinoma of the stomach: Areas of failure in a re-operation series (second or symptomatic look). Clinicopathologic correlation and implications for adjuvant therapy. *Int J Radiat Oncol Biol Phys* **8:** 1–11

Hagiwara A, Takahashi T, Kojima O, Sawai K, Yamaguchi T, Yamane T, Taniguchi H, Kitamura K, Noguchi A, Seiki K and Sakakura C (1992) Prophylaxis with carbon-adsorbed Mitomycin against peritoneal recurrence of gastric cancer. *Lancet* **339:** 629–631

Landry J, Tepper JE, Wood WC, Moulton EO, Koerner F and Sullinger J (1990) Patterns of failure following curative resection of gastric carcinoma. *Int J Radiat Oncol Biol Phys* **19:** 1357–1362

Markman M (1991) Intraperitoneal Chemotherapy. *Semin Oncol* **18:** 248–254

McCulloch P (1995) Extended lymphadenectomy for gastric cancer. *GI Cancer* **1:** 105–112

Shah IA, Lindup WE and McCulloch P (1997) Variability of Mitomycin C adsorption to activated charcoal. *Br J Cancer* **75:** 13

Siewert JR, Bottcher K, Roder JD, Busch R, Hermanek P and Meyer HJ (1993) Prognostic relevance of systemic lymph node dissection in gastric carcinoma. *Br J Surg* **80:** 1015–1018

Takahashi T, Hagiwara A, Shimotsuma M, Sawai K and Yamaguchi T (1995) Prophylaxis and treatment of peritoneal carcinomatosis: Intraperitoneal chemotherapy with Mitomycin C bound to activated carbon particles. *World J Surg* **19:** 565–569

© Cancer Research Campaign 1997